# Mitochondrial DNA and Neurodegeneration: Any Role for Dietary Antioxidants?

**DOI:** 10.3390/antiox9080764

**Published:** 2020-08-17

**Authors:** Laura Bordoni, Rosita Gabbianelli

**Affiliations:** Unit of Molecular Biology, School of Pharmacy, University of Camerino, 62032 Camerino, Italy; rosita.gabbianelli@unicam.it

**Keywords:** nutrigenomics, mitochondria, epigenetics, antioxidants, neurodegeneration, inflammation, redox system, oxidative stress, diet, nutrition

## Abstract

The maintenance of the mitochondrial function is essential in preventing and counteracting neurodegeneration. In particular, mitochondria of neuronal cells play a pivotal role in sustaining the high energetic metabolism of these cells and are especially prone to oxidative damage. Since overproduction of reactive oxygen species (ROS) is involved in the pathogenesis of neurodegeneration, dietary antioxidants have been suggested to counteract the detrimental effects of ROS and to preserve the mitochondrial function, thus slowing the progression and limiting the extent of neuronal cell loss in neurodegenerative disorders. In addition to their role in the redox-system homeostasis, mitochondria are unique organelles in that they contain their own genome (mtDNA), which acts at the interface between environmental exposures and the molecular triggers of neurodegeneration. Indeed, it has been demonstrated that mtDNA (including both genetics and, from recent evidence, epigenetics) might play relevant roles in modulating the risk for neurodegenerative disorders. This mini-review describes the link between the mitochondrial genome and cellular oxidative status, with a particular focus on neurodegeneration; moreover, it provides an overview on potential beneficial effects of antioxidants in preserving mitochondrial functions through the protection of mtDNA.

## 1. Introduction

Neurodegenerative diseases are characterized by the progressive degeneration of neurons and synapses of the brain cortex and some subcortical regions in the central nervous system. This process can be induced by various neurotoxic events, such as excessive inflammation, ROS production, and mitochondrial dysfunctions, among many others. 

Indeed, due to their limited glycolytic capacity and extremely metabolically active nature, neurons are energetically demanding cells [1]. Since the energetic balance in nucleated eukaryotic cells strongly depends on mitochondria, neuronal health and survival require a delicate maintenance of the mitochondrial function. Mitochondria are cytoplasmic organelles that play a central role in energy generation by oxidative phosphorylation (OXPHOS) that occurs in the mitochondrial cristae (inner membrane’s convolutions) through five protein complexes (I–V). During OXPHOS, these complexes work along with electron carriers as an electron transport chain, also known as respiratory chain, receiving and donating electrons to the next complex until reaching complex V [2]. However, mitochondria do not only produce cellular adenosine triphosphate (ATP), but also regulate programmed and unprogrammed cell death, Ca^2+^ hemostasis and, especially, generate endogenous reactive oxygen species (ROS), whose overproduction may cause further damage to mitochondrial components and promote disease development. Coherently, a large body of evidence suggests that mitochondrial dysfunctions are prominent early features of neurons affected by neurodegenerative diseases [3,4]. 

Like for many progressive and multifactorial pathologies, environmental exposures strongly modulate the risk for these diseases’ onset, even in presence of a genetic predisposition. Since mitochondria are exquisitely sensitive to environmental threats, they act at the interface between inner and outer cellular environment. Hence, given the pivotal role of the mitochondrial function and ROS overproduction in the pathogenesis of neurodegeneration [5], antioxidants have been suggested to counteract the detrimental effects of ROS in neurodegenerative disorders [6,7]. In particular, mitochondria-targeted antioxidant therapies are suggested as promising strategies to promote mitochondrial function and neuronal health [8,9,10]. 

Remarkably, mitochondria are unique organelles since they contain their own genome (mitochondrial DNA, mtDNA), which directly regulates mitochondrial functions. Indeed, it has been highlighted that mtDNA genetics but also epigenetics might play relevant roles in modulating the risk for neurodegenerative disease, acting at the interface between environmental exposures and the molecular triggers of neurodegeneration. This review describes the link existing between the mitochondrial genome and cellular oxidative status, with a particular focus on neurodegeneration. Moreover, it discusses the potential beneficial effects of antioxidants in preserving the mitochondrial function through the protection of mtDNA genetics and epigenetics.

## 2. Mitochondrial DNA: From Genetics to Epigenetics

The human mtDNA is a double-stranded, circular molecule of 16,569 bp, where one strand is purine rich (i.e., heavy strand) and the complementary strand is rich in pyrimidines (i.e., light strand) [11]. Only about 3% of the mtDNA is noncoding (containing two replication origins, O_H_ and O_L,_ on the heavy and light strands, respectively), and the coding sequences can be transcribed on both DNA strands in a polycistronic manner into a large mitochondrial mRNA. The 37 genes contained in the mtDNA encode for 13 polypeptides (all subunits of enzyme complexes of the oxidative phosphorylation system), 22 transfer RNAs (tRNAs), and 2 ribosomal RNAs (rRNAs), required for the translation of each subunit within the mitochondrial compartment [12]. MtDNA regulates several, but not all, mitochondrial functions. Indeed, the mtDNA undergoes an intense crosstalk with the nuclear DNA (nDNA), which also regulates numerous mitochondrial tasks [13,14]. 

It is of common knowledge that mtDNA is maternally inherited. In fact, despite some evidence of a biparental inheritance of mtDNA having been proposed [15], this hypothesis lacks definite confirmation [16,17,18,19]. 

Notably, mitochondria are highly dynamic organelles: they undergo regular fusion, fission, and transport, the processes of which determines mitochondrial location, morphology, and number [20]. These processes are linked to the mitochondrial genome [21], which can be replicated and translated, and thus regulate the number and the functionality of mitochondria.

Typically, multiple copies of the mtDNA populate each individual mitochondrion and hundreds to thousands of mtDNA copies may be present in a single cell (i.e. polyploidy). Due to the presence of multiple copies of the mtDNA per cell, different alleles or mutations (either inherited or sporadically occurred) can be present in only a fraction of the total pool of mtDNA in a given cell or organism (i.e., heteroplasmy) [22]. Different levels of heteroplasmy may exist between tissues and cells of the same individual, resulting in complex phenotypic manifestations [23].

The mtDNA has a high mutation rate, and numerous mutations have been linked to a range of diseases [24,25]. In parallel, several mutations of nuclear genes essential for mtDNA maintenance have been identified as cause of mtDNA point mutations, multiple deletions, or mtDNA depletion [26].

In recent decades, it has been suggested that not only mtDNA genetics but also epigenetics can regulate mitochondrial functions. This regulation is called mitoepigenetics [27,28]. Since the mtDNA lacks histones (it is packaged by the mitochondrial transcription factor A, TFAM, into nucleoprotein complexes known as nucleoids), the focus has been mainly centered on DNA methylation. However, post-translational modification of the TFAM and non-coding RNAs can also contribute to regulation of gene expression and mtDNA replication [29]. The existence of an epigenetic regulation of mtDNA and its role have been heavily debated, particularly with regards to DNA methylation. In mtDNA, methyl groups donated from S-adenosyl-methionine (SAM) can be added to cytosines (5mC, 5-methyl cytosine) or adenine (6mA, 6-methyl adenine) by DNA methyltransferases enzymes (e.g., DNA methyl-transferase 1, DNA methyl-transferase 3a, and DNA methyl-transferase 3b) which have been identified also within mitochondria [30,31], suggesting that methylation occurs in mtDNA in CpGs but also in non-CpG contexts [32]. Also ten-eleven translocation proteins (TET1 and TET2) have been detected in the mitochondria [33], indicating that mtDNA demethylation can occur both via passive mechanisms or active oxidation-mediated demethylation [29,34,35]. An area of the mtDNA that has been frequently investigated for changes in methylation is the three-stranded displacement (D-loop) structure, that is located in the noncoding control region and regulates transcription and replication of mtDNA. The human mitochondrial D-loop region has 1122 base pairs, and its methylation may have a direct role in controlling mtDNA replication [36] and, possibly, transcription [37]. Indeed, since mitochondrial DNA polymerase γ requires an RNA primer, mtDNA replication is tightly linked to transcription [38]. Indeed, the D-loop locus implicated in the processing of the RNA primer for mitochondrial replication was found to be methylated in human and murine peripheral blood [33,39], suggesting a role for mtDNA methylation in the control of mtDNA replication [40]. Indeed, D-loop methylation alterations have been associated with numerous pathological conditions, ranging from cancer to CVD, neurodegeneration, and others [36,39,41,42,43,44].

An additional level of gene regulation might be provided by TFAM acetylation/phosphorylation, which modulates compaction of mtDNA into nucleoids and is thought to be crucial for regulating mtDNA segregation and expression. In particular, it has been shown that acetylation and phosphorylation of TFAM can fine-tune TFAM–DNA binding affinity to permit the discrete regulation of mtDNA dynamics. In particular, phosphorylation of TFMA has been demonstrated to regulate transcription by altering the ability of TFAM to locate promoter sites [45].

Furthermore, noncoding RNAs can also act at the mitochondrial level [29]. Indeed, mature micro-RNAs (miRNAs) are commonly present in the cytosol of cells, and it has been widely established that they are stable also in biological fluids (characteristic that makes them attractive biomarkers). Various studies have revealed that miRNAs can act also within the mitochondria (mito-miRNAs). In particular, mitomiRs are implicated in inflammaging [46], influencing the energetic, oxidative, and inflammatory status of senescent cells. These mito-miRNAs include miR-130a-3p [47], let-7b [48], miR-146a-5p [49], miR-181c-5p [50], miR-133a, and miR-1 [51]. Moreover, it has been demonstrated that they are differentially expressed in several pathologies [52], ranging from cardiovascular [53] to neurodegenerative diseases [54]. Hence, they are supposed to play a role in regulating mitochondrial gene expression and functions in both physiological and pathological conditions. However, despite changes of mito-miRNAs’ expression having been widely demonstrated, mechanistic details on their mode of action in the mitochondria are not clearly established. Most of the mitomiRs are encoded by the nuclear genome and translocate to the mitochondria [55], while some others can be encoded directly by the mitochondrial genome [56].

Furthermore, given the proximity of mtDNA to the sites of oxidative phosphorylation at the inner membrane of the mitochondria, it is not surprising that mtDNA is especially prone to oxidized DNA damage. As a result, oxidative damage and loss of mtDNA integrity are increasingly recognized to play a role in the development of many chronic age-related diseases [57]. Not only the protection from oxidative damage, but also the regulation of mtDNA copy number (although apparently in excess) has been revealed to be an important aspect of mitochondrial biogenesis, essential for normal cellular functions [58,59].

Therefore, the control of mtDNA replication, transcription and repair in the face of constant onslaught from endogenous and environmental agents has a central role in health [60,61]. Indeed, it has been demonstrated that environmental exposures, contributing to the onset of new mutations or oxidative damages or modulating epigenetic features of mtDNA, strongly impact mitochondrial health. In the light of this evidence, mtDNA depletion and malfunction have been implicated in cancer [62,63], metabolic and cardiovascular diseases [43,64,65,66,67], aging [68], and many human multifactorial disorders, including neurodegeneration [69,70].

## 3. Mitochondria in Neurodegeneration: The Interplay between Oxidative Stress and the Mitochondrial Genome

Neuron cells are particularly vulnerable to oxidative damage because of several reasons. Their membranes are rich in polyunsaturated fatty acids (PUFAs), that can be easily oxidized. They also contain abundant redox-active proteins with metals (e.g., iron, copper, etc.) as prosthetic groups, which can play a role in ROS metabolism [71,72]. The high oxygen consumption typical of these cells increases the risk of oxidative neuronal damage in particular at mitochondrial level, where oxygen is reduced to water during the oxidative phosphorylation. Indeed, at rest, about 1% of the oxygen consumed is partially reduced to superoxide anion in mitochondria of adult cells, and then reduced by the antioxidant system [73]. Hence, any deficiency in the antioxidant defense system that reduces ROS levels makes the brain more vulnerable to oxidative stress [5].

The oxidative stress resulting from both increased ROS production and/or the inadequacy of the antioxidant system’s response can lead to protein, lipid, and DNA oxidation. Interaction with oxygen atoms can induce oxidation of DNA bases, in particular guanine, which has the lowest reduction potential of the four DNA bases [74]. Guanine interacts with hydroxyl radicals and generates one of the major products of DNA oxidation, such as 8-oxo-2′-deoxyguanosine (8-oxo-dG), whose levels are increased in neurodegenerative disorders [75]. PUFAs located in the phospholipid bilayers are easily oxidized by ROS into fatty acid radicals. These unstable fatty acid radicals interact with oxygen, thus producing fatty acid peroxyl radicals, which interact with other PUFAs and trigger the production of other fatty acid radicals and lipid hydroperoxides. This repeated cycle of lipid peroxidation strongly affects structure and functions of biological membranes [76]. Moreover, it is well-known that ROS can also damage proteins by oxidation of amino acids, especially cysteine and methionine, thus triggering protein modifications—such as protein fragmentation by oxidation of the protein backbone, protein–protein cross-linking, amino acid side chain modification, and enzyme inactivation—resulting in activity loss [77]. 

Since the mitochondria are the center of metabolic pathways (i.e., citric acid cycle, fatty acids metabolism, urea cycle, etc.), increased mitochondrial ROS production can perturb the activity of enzymes involved in these paths, affecting both the ATP production and the metabolic responses. Given the previously explained effects of ROS on biological molecules, mitochondrial membranes are also susceptible to ROS oxidation, especially at the level of proteins and unsaturated fatty acids [78]. In particular, oxidation at the unsaturated acyl chains modifies the intermolecular interactions, changing the membrane fluidity and indirectly affecting protein functions. In addition, oxidation induces the formation of weak bonds between functional groups of proteins and other oxidized molecules, hence decreasing their dynamicity. Moreover, ROS control the expression of highly conserved heat-shock proteins, and the increased mitochondrial ROS production can perturb the folding of proteins synthesized by mitochondria [79]. Finally, oxidation of cardiolipin‘s acyl chains at the mitochondrial inner membrane can cause respiratory complex destabilization [79,80]. Cardiolipin can interact with the respiratory chain complexes and contribute to generate the electrochemical gradient. Additionally, since it can interact with death-inducing proteins, it is involved in the apoptotic process and oxidation of cardiolipin compromises apoptosis, promoting neuronal dysfunctions and the progression of neurodegeneration (e.g., Alzheimer’s and Parkinson’s disease) [80,81]. Coherently with this picture, plenty of studies highlight the involvement of mitochondrial dysfunctions in neurodegeneration [3,82,83,84], supporting the relevance of protecting systems, including antioxidant defenses, to maintain neuronal integrity and survival [85].

At the interface between oxidative stress and the mitochondrial function there is also the mtDNA. Indeed, it has been demonstrated that inhibition of mitochondrial dynamics affects the elimination of mtDNA damage and the transmission of mtDNA mutations [86,87]. DNA damage is a well-established trigger of apoptotic cell death in mitotic cells as well as in terminally differentiated cells such as neurons [88]. However, DNA repair enzymes can prevent the accumulation of mutation, and this has been well described in the nuclear DNA. Differently, mechanisms of damage and repair in the mitochondrial genome are poorly understood, despite the fact that mtDNA is subjected to higher levels of oxidative stress than is nuclear DNA [89,90,91]. This particular sensitivity to oxidative agents is likely due, as previously mentioned, to its proximity to the inner mitochondrial membrane, where oxidants are formed, but also to the lack of protective histones which lower the frequency of DNA strand breaks by scavenging the hydroxyl radicals [92,93]. Indeed, it has been proven that impairments in mitochondrial respiration and oxidative phosphorylation elicit an increase of mtDNA rearrangements and mutations [94], which has been linked to neurodegeneration [95,96,97]. On the other hand, it has been also demonstrated that mutations in the mtDNA exacerbate ROS production, contributing to disease onset and progression [98]. Moreover, alterations of the mitochondrial genome in neurodegeneration have been demonstrated also in terms of variation of copy number [99], that significantly varies in neurodegenerative diseases in respect to healthy controls, and has been proposed as an innovative biomarker [100,101,102,103]. This evidence supports the hypothesis that the mitochondrial genome significantly influences both pathological (e.g., neurodegeneration) and physiological processes (e.g., aging) [104]. 

Recently, in particular for neurodegenerative disorders, the new field of mitoepigenetics is grabbing a growing attention [6,40,42,105]. The role of epigenetic modifications in neurodegeneration has been widely discussed and recognized [106,107,108,109,110] both in animal models and humans [108,111,112,113,114], in particular in the case of early-life exposure to xenobiotics [110,115,116]. Nuclear epigenetics can drive the expression of genes involved in mitochondrial homeostasis, and, conversely, metabolic alterations occurring in mitochondria may affect the availability of substrates for epigenetic enzymes [117], thus leading to alterations of epigenetic signatures in the nuclear genome [13,117]. Currently, a large body of evidence supports the existence of a direct epigenetic regulation of mtDNA, though the mechanistic effect of epigenetic marks on gene expression in mitochondria is still debated [37,117,118,119]. Remarkably, impaired methylation levels of the D-loop region in animal models, postmortem brain regions, or circulating blood cells of patients with Alzheimer’s disease, Parkinson’s disease, and amyotrophic lateral sclerosis, suggested that alterations of epigenetic regulations of mtDNA may mediate mitochondrial dysfunctions that are typical of neurodegenerative disorders [40]. Furthermore, the possibility that mtDNA methylation could act as a mediator between environmental exposure and the mitochondrial genome [120] might provide further insights on the complex environmentally linked etiology of neurodegenerative disorders. Finally, since epigenetic therapies have been proposed for treating neurodegenerative disorders [121,122], elucidating the effect of epigenetic regulations on the mitochondrial DNA might provide additional information in view of the future application of epigenetic therapies in clinical practice. 

## 4. Antioxidants, Gene Expression, and Epigenetic Regulations: The Role of Nutriepigenomics in Neurodegenerative Diseases 

Appropriate nutrition is an essential and modifiable factor that plays a key role in preventing and/or delaying the onset of neurodegeneration. Reducing risk factors linked to the lifestyle (i.e., smoking of cigarettes, limiting stress, avoiding toxins, promoting mental and physical training) but also preventing hypertension, type 2 diabetes, insulin resistance and obesity, increase of homocysteine, are important components of neurodegenerative disease prevention. For these reasons, the early protection from neurodegeneration starts with the choice of appropriate types and quality of food [110,123,124,125]. Indeed, positive clinical outcomes exerted by antioxidants supplementation. For example, vitamins C [126,127], E [128] and N-acetylcysteine [129] have been demonstrated active in oxidative-stress related pathologies [130,131]. A large body of evidence shows that many dietary antioxidants can help to delay their onset and progression [6,132,133,134], in particular when applied at the early-stages of the disease, and the application of dietary antioxidants for the prevention of neurodegeneration has also been proposed [71].

From the molecular point of view, numerous pathways are triggered by an appropriate nutrition, and a clear role of nutrigenomics in promoting neuronal health has been demonstrated [135]. Several vegetable foods rich in polyphenols (e.g., resveratrol [136], carotenoids [137], quercetin [138,139]) and other bioactive compounds (e.g., sulforaphane [140,141,142], melatonin [143], docosahexanoic acid [144], etc.), have been shown to be efficacious in preventing neurodegeneration by regulating gene expression and epigenetic regulation of anti-inflammatory and antioxidant pathways [110,135,145]. Studies on experimental models of neurodegeneration demonstrate that early treatment with antioxidants (i.e., coenzyme Q10, vitamin E, vitamin C) can prevent the progressive neuronal damage by reducing neuroinflammation, which is associated with increased production of proinflammatory cytokines (i.e., TNF-a, Rantes). These antioxidants counterbalance the progression of oxidative damage at protein, lipid and DNA level, as well as counteracting the decrease of glutathione (GSH) in the striatum [133,146]. These antioxidants also modulate the expression level of the pro-inflammatory nuclear factor kappa-light-chain-enhancer of activated B cells (NFkB), as well as of the anti-inflammatory nuclear factor erythroid 2-related factor 2 (Nr-f2) [110]. In vitro studies on dopaminergic cell line show that GSH and tocotrienol can actively prevent oxidative neuronal damage [133,147]. Sulforaphane, contained in broccoli, modulates the antioxidant and anti-inflammatory responses by modulating the nuclear erythroid 2-related factor 2/ antioxidant response element (Nrf2/ARE) pathway, inhibiting NFkB and promoting cell detoxification by stimulation of phase II enzymes, that increase xenobiotics hydrophilicity and enhance their consequent excretion [142]. Besides, sulforaphane reduces the β-amyloid (Aβ) protein burden by upregulation of p75 neurotrophin receptor and histone deacetylases’ (HDAC1 and HDAC3) expression in an Alzheimer’s disease animal model [148]. Resveratrol is able to prevent microglia activation in Alzheimer’s disease due to its anti-inflammatory activity. It increases anti-inflammatory cytokine release and upregulates sirtuin 1 (SIRT1) expression which, due to its deacetylase activity, exerts a neuroprotective action [149,150]. In addition, resveratrol activates 5′-adenosine monophosphate-activated protein kinase (AMPK)-dependent signaling saving Aβ-mediated neurotoxicity in human neural stem cells [151]. Curcumin can cross the blood–brain barrier where it exerts a protective role against neurodegeneration in both Parkinson’s and Alzheimer’s diseases. In vitro and in vivo study show that curcumin has antioxidant and anti-inflammatory properties, together with the capacity to limit protein aggregation (i.e., a-synuclein, amyloid beta peptide, hyperphosphorylated tau) [152].

Moreover, an anti-inflammatory and antioxidant diet (especially during the early-life but also later) represents a central strategy for modulating brain plasticity and building an “epigenetic memory” that might promote neuronal resilience against stressors and prevent neurodegenerative phenomena [110,135]. Indeed, oxidative stress can also directly and indirectly influence the cellular epigenetic homeostasis [153,154]. Both DNA oxidation and TET-mediated hydroxymethylation affect the methylome [155]. ROS can directly convert 5mc to 5-hydroxymethylcytosine (5hmC) [156], that affects DNA methyl-transferase 1 (DNMT1) activity, thus leading to an improper methylation inheritance during mitosis and global hypomethylation [157]. Furthermore, they can modulate the methylome through the oxidization of guanosine to 8-oxo-20-deoxyguanosine (8-oxodG) thus inhibiting methylation of adjacent cytosine and further contributing to global hypomethylation of DNA [158,159]. At the same time, intracellular levels of metabolites such as acetyl-coenzyme A (acetyl-CoA), Fe, ketoglutarate, NAD+, and S-adenosylmethionine (SAM), which are essential for the epigenetic machinery (in particular for the activity of histone-modifying enzymes), depends on the global cellular metabolism and energy levels of the cell, thus they are strictly linked to the cellular oxidative status [160,161,162]. A tight interplay exists also between oxidative stress and miRNAs expression levels, since ROS affect miRNAs expression [163] and, conversely, miRNAs regulate the expression of numerous genes involved in an oxidative stress response [164,165]. Therefore, oxidative stress can globally influence the cell on multiple levels, including its epigenetic landscape [166], and can contribute to the pathogenesis of neurodegenerative disorders [111,167,168]. Given the crucial role of nutrition in the maintenance of the epigenetic regulation [169], and given the effects of dietary antioxidant in the modulation of the redox environment in the cell, an antioxidant and epigenetic diet contributes to preservation of cellular homeostasis [169] in both health and disease [170].

The existence of a link between nutrition and epigenetic reprogramming is well known [169]. Dietary intake of folate, vitamins B12, B2, B6, choline, betaine, and methionine impact the one carbon cycle and the cellular bioavailability of S-adenosylmethionine, which is the universal methyl-donor [169]. In addition, the mitochondrion, via its nucleo-cytoplasmic pools, is a major regulator of cellular levels of acetyl-CoA, the main acetyl donor [171]. Moreover, bioactive molecules contained in food [172], total caloric intake and specific dietary regimens [173] can also modulate the epigenome. Indeed, nutrition can affect the epigenome through at least two general mechanisms: either directly, by providing molecules which interact with the enzymes responsible for ‘writing’ or ‘erasing’ the epigenetic profiles, or indirectly through metabolic rewiring. Remarkably, both mechanisms converge on mitochondria, which produce a plethora of factors and substrates essential for epigenetic modifications and are at a crossroads of cellular energy metabolism [174].

Evidence about correlations between dietary antioxidants and epigenetic regulations in the nDNA and the associated molecular mechanisms has been recently and comprehensively reviewed by Beetch et al. [175]. It has been observed that compounds that decrease oxidative stress consequently impact DNA methylation landscapes. Despite most of evidence coming from in vitro or animal studies, vitamin C [176,177,178], A [179,180,181], and E [182], selenium [183], stilbenoids [184,185], epigallocatechin gallate [186], genistein [187], curcumin [188], and sulphoraphane [189,190] are all molecules that have been shown to mediate both epigenetic regulations and, as previously described, oxidative stress, demonstrating that these two pathways in the cell are strictly related [175]. Also curcumin has nutriepigenomics properties because it can inhibit DNA methyltransferases and regulate chromatin condensation by modulating histone acetyltransferases and histone deacetylases [191].

Summarizing, several dietary components and bioactive molecules can be used to contrast neurodegeneration due to their capacity to modulate oxidative stress, gene expression, and epigenetics regulations. Their employment has to be regular and in the first stages of neuronal damage. 

## 5. Antioxidants and Mitochondria: From the General Function to the Mitochondrial Genomics

For a long time, a wide consensus has been given to the mitochondrial free radical theory of aging [192], which asserts that, by damaging mitochondria, external ROS can promote mtDNA damages and increase the endogenous production of free radicals, promoting mitochondrial dysfunctions. Thus, the usage of antioxidants and bioactive compounds that might act at the mitochondrial level has been proposed also in neurodegeneration to overcome excessive ROS production. For example, the manganese superoxide dismutase (MnSOD), encoded by genomic DNA, protects mitochondria from oxidative stress. This enzyme, located in the mitochondrial matrix, catalyzes the dismutation of superoxide anion, generated during the monoelectronic reduction of oxygen to water, into hydrogen peroxide. Clinical findings show that, in the early stage of neurodegeneration, a compensatory upregulation of MnSOD can be observed [193,194,195,196]. Oxidative stress upregulates MnSOD and its enzymatic activity can be modulated by several bioactive compounds (e.g., melatonin, dimethyl fumarate, etc.) and dietary antioxidants (e.g., tea catechin, curcumin, etc.). Melatonin promotes neuroprotection of Purkinje cells by MnSOD upregulation [197], as has been demonstrated for dimethyl fumarate in SH-SY5Y human neuroblastoma cell lines [198]. Longjing green tea catechin extract significantly upregulates MnSOD and increases its enzymatic activity in *Drosophila melanogaster* [199]. Curcumin can reduce mitochondrial superoxide anion accumulation and downregulate MnSOD in HT22 cells [200,201]. Pomegranate-derived polyphenols exert anti-inflammatory, antioxidant, and antimicrobial activity [202]. The pomegraniin A contained in the fruit extract reduces ROS by MnSOD activation. An increase of ROS production stimulates mitochondrial deacetylase sirtuin 3 (SIRT3) leading to MnSOD activation [203,204]. Quercetin supplementation prevents the aluminum-induced neurodegeneration in animal model by increasing MnSOD activity and glutathione levels and reducing lipid and protein oxidation [205]. Summarizing, the upregulation of MnSOD in the mitochondria exerted by antioxidants can help to counterbalance the disequilibrium in the redox homeostasis, preventing the ROS-induced neuronal damage and the progression of the neuronal disease.

Many of the previously mentioned bioactive molecules, which have both antioxidant and nutrigenomic effects, act at the interface with the mitochondrion [174]. Dietary supplements that can help to maintain mitochondrial homeostasis include, among others, coenzyme Q10, vitamins C, E, K1 and B, L-carnitine, and other mitochondrion-targeted antioxidants such as N-acetylcysteine, sodium pyruvate, and lipoic acid [206]. Also, flavonoids such as ampelopsin and pinocembrin can inhibit mitochondrial dysfunction and neuronal death through the regulation of gene expression of Nrf2 pathway. Moreover, supplementation with resveratrol also increases mitochondrial mass. It mediates the SIRT1-dependent deacetylation of peroxisome proliferator-activated receptor gamma coactivator 1-alpha (PGC-1α), thus resulting in stimulation of the mitochondria biogenesis, which in turn co-activates the nuclear respiratory factors (NRF-1 and NRF-2), that also promote the mitochondrial biogenesis [207,208,209]. Despite a lack of consensus about which is the general mechanism of action that could explain these effects induced by the resveratrol on mitochondria, some hints suggest the involvement of mitochondrial complex III [209,210]. Similarly, a role for mitochondria on the anti-inflammatory effects of curcumin has been also hypothesized, since a homozygous deletion of the mitochondrial uncoupling protein 2 is able to reverse the beneficial effects of this molecule in mice [211]. In addition, genistein can modulate the enzymatic activity of components of the oxidative phosphorylation system [174]; isoflavones can stimulate mitochondrial biogenesis and improves mitochondrial function in diabetes, as well as in aging of rodents [212,213]; sulphoraphane can enhance ROS and mitochondrial membrane depolarization in human ovarian cancer cell lines [214]. Interestingly, caloric restriction has been shown to exert many healthy effects (including neurodegeneration prevention [215,216]), consistent with reduced ROS generation by mitochondria, and many antioxidant compounds can also mimic such effects. For examples, N-acetyl cysteine (NAC) provides thiol groups to glutathione and to mitochondrial respiratory chain proteins. Thus, it may counteract both ROS generation and effects [217,218]. Of note, it has been widely demonstrated that the beneficial effects of caloric restriction are also mediated by epigenetic mechanisms [219,220], that, as previously described, are tightly linked with the oxidative status of the cell.

However, it must be also considered that ROS are generated by numerous biochemical reactions in the cell and have not only detrimental but also physiological roles. The general antioxidants do not have a selective action, and these substances are often used at quite high concentrations in order to obtain a therapeutic effect. Thus, mitochondria-targeted antioxidants has been proposed as a strategy to boost mitochondrial biogenesis and organelle bioenergetics, limiting mitochondrial ROS production and oxidative damage, without suppressing ROS that are important for cell signaling [221,222,223]. Promising results has been obtained on mitochondrial biogenesis and functions with this kind of specific antioxidants, including improving insulin sensitivity, muscle contractile function, and sarcopenia [224]. In particular, one of the most promising mitochondria-targeted antioxidant is the mitoquinone, or Mito-Q. Mito-Q consists of a quinone moiety linked to a triphenylphosphonium (TPP) moiety by a 10-carbon alkyl chain [225]. It is several-hundred-fold more potent than untargeted antioxidants in blocking ROS and preventing mitochondrial oxidative damage [226]. It is orally active, can cross mammalian membranes and has been revealed particular effective against lipid peroxidation [227]. Remarkably, it displayed a neuroprotective effect that make it a potential candidate against neuroinflammatory diseases, such as neurodegenerative disorders [228]. Additionally, it has been demonstrated to be effective also in the treatment of peripheral neuropathy in animal models of diet-induced obesity, increasing the possibility of its application in clinical practice [229].

Furthermore, taking into account the physiological role of ROS production in the cell, it has been recently suggested that mtDNA mutations may be generated by replication errors rather than by accumulated oxidative damage, so that mitochondrial dysfunction due to mtDNA alteration may rather be the major driver of the aging process [98,230]. This theory overturns the free-radical theory of aging, suggesting that the increase in ROS might be a consequence rather than a cause of aging and that ROS are mediators of the stress response to age-dependent damage [230,231]. This vision strengthens the hypothesis of a pivotal role of mitochondrial genome in driving pathological and physiological processes and might be reinforced also by the new discoveries on mitochondrial epigenetics. The interaction between mtDNA and oxidative stress have been proved also by demonstrating that increased mitochondrial oxidative stress derived from MnSOD mutant mice (Sod2−/+) promoted mtDNA glycation [232] and a 30% increase in the 8-OH deoxyguanosine levels of mtDNA [233], suggesting that aberrant regulations of this enzyme can affect also the mitochondrial DNA. On the other hand, unexpected evidence has been collected about absence of correlation between MnSOD activity and mtDNA mutation rate, that did not vary significantly in loss of function mutants in respect to controls [234,235]. 

Several bioactive molecules can act through interaction with the mtDNA. For example, CoQ10 diet is able to preserve mtDNA content and Tfam/OXPHOS complex protein expression in a mouse model of glaucoma [236]. In a case report, CoQ10 deficiency has been associated with a mitochondrial DNA depletion syndrome in a human case [237]. Remarkably, the mitochondrially-targeted Mito-Q has been demonstrated to protect the intestinal barrier by ameliorating mitochondrial DNA damage via the Nrf2/ARE signaling pathway [238]. Resveratrol can restore the suppressed SIRT1 expression and mtDNA expression in a cellular model of ovarian hypoxia [239]. Flavanones (in particular, naringin) are able to modulate mtDNA copy number in human leukocytes and has been identified as the key factor behind the association between fruit consumption and mtDNA copy number measured in a population based study [240]. Genistein alleviates the mitochondria-targeted DNA damage induced by β-amyloid peptides 25–35 in C6 glioma cells [241]. In particular, levels of 8-OHdG and mtDNA deletion decreased after a pre-treatement with genistein. Curcumin is able to suppress gastric tumor cell growth via decreased mtDNA content and ROS-mediated DNA polymerase γ depletion, thus reducing mitochondrial oxygen consumption and aerobic glycolysis [242]. In a rat model of myocardial ischemia reperfusion, EGCG may mediated cardioprotective effects also by inhibiting the release of mtDNA from damaged mitochondria, which have been proven to be a potent pro-inflammatory mediator [243]. Furthermore, EGCG may exert anti-obesity properties also by improving thermogenesis and mitochondria biogenesis in the brown adipose tissue (BAT). Indeed, EGCG-fed mice displayed higher body temperature and mtDNA content in BAT [244]. Of note, not only dietary antioxidants but other nutrients [245] or dietary regimen [246,247], as well as alteration of body composition [65], has shown association with mtDNA copy number, corroborating the hypothesis that diet and health status are strictly linked with the mtDNA function. Conversely, it has been recently demonstrated that alteration of mitochondrial DNA content can modulate antioxidant enzyme expressions and, consequentially, oxidative stress in the cell [248].

Mitochondria are also important for epigenetic regulations since these are strictly linked to the bioenergetic system, which provides the interface between the environment and the epigenome by converting environmental calories into ATP, acetyl-CoA, SAM, and reduced nicotinamide adenine dinucleotide (NAD+). Consistently, the clinical phenotypes of bioenergetic diseases are quite similar to those observed in epigenetic diseases (e.g., Rett, Angelman, Fragile X Syndromes, etc.), and an increasing number of epigenetic diseases are being associated with mitochondrial dysfunction [249]. Thus, the study of this bioenergetic–mitochondria–epigenomic network has broad implications for identifying the etiology, pathophysiology, and treatment of a wide range of common diseases.

While the correlation between nutrition, oxidative stress, and the nuclear epigenome is well described, to our knowledge, there are no molecular data on the effects of general antioxidants or mitochondria-targeted antioxidants on mtDNA epigenetics as yet. However, a certain responsiveness of the mitochondrial epigenome to external stimuli, including but not limited to dietary elements, has been postulated [65,67,120]. Remarkably, Corsi and colleagues recently demonstrated that mtDNA methylation can change accordingly to the adherence to the Mediterranean diet [250], corroborating the hypothesis of a potential role of nutrition in the modulation of mtDNA methylation.

In conclusion, although potential applications of this research field are wide, it must be considered that the body of literature investigating molecular effects of bioactive compounds and dietary antioxidants on the mtDNA are still mainly based on in vitro or in vivo studies. Few data have been collected in population-based studies involving dietary supplementations. For this reason, randomized clinical trials or well-controlled observational studies that could confirm or rather reject the evidence from current basic research are necessary before considering applications in clinics. In particular, only a limited number of studies have investigated mitoepigenetic changes in humans or animal models of neurodegenerative disorders so far. Hence, despite encouraging preliminary data, further molecular and functional studies are required to elucidate the impact of mtDNA methylation on the mitochondrial function in neurodegenerative diseases. 

Moreover, concerning the field of mitochondrial epigenetics, the existence of this additional level of regulation on mtDNA is still questioned by some authors [251,252]. There are also discrepancies about where methylation occurs in mtDNA [32,37] and what methods and technologies should be used to study this mark [35,253]. It is still to be clarified, above all, what are the mechanistic aspects through which alterations of the mtDNA epigenome can lead to mitochondrial dysfunctions. Indeed, the mechanism through which mtDNA methylation is regulated and regulates mtDNA replication and transcription is under investigation [32,37,119,253,254]. Since there are still many aspects to be clarified, mitochondrial epigenetics represents a growing but also discussed research area that needs further confirmation to be definitely established and translated into clinical practice.

**Figure 1:**
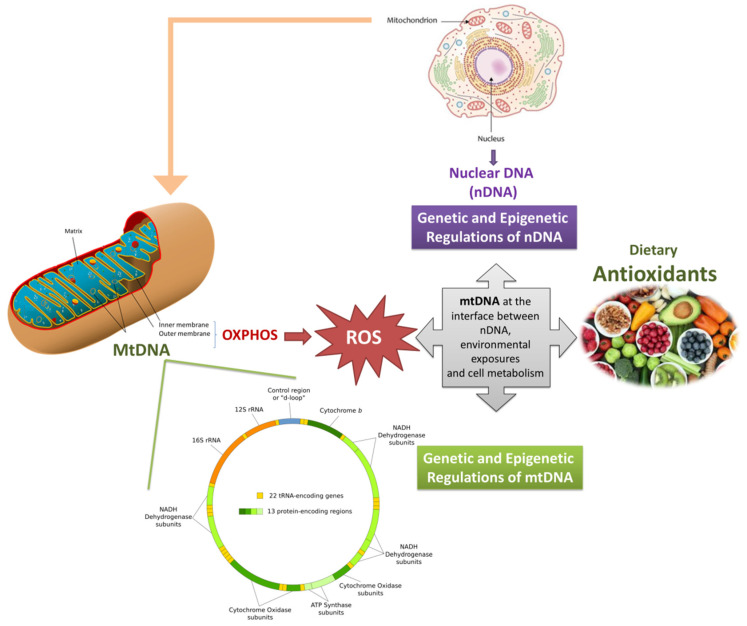
Graphical representation of the interplay between mitochondrial genome (and epigenome), dietary antioxidants, and mitochondrial health. Mitochondria produces energy but also ROS, whose overproduction might induce mitochondrial dysfunctions, also through the alteration of the mitochondrial genome. Dietary antioxidants, can promote the mitochondrial function by reducing ROS production, modulating epigenetic pathways and, thus, restoring cellular homeostasis. Since mitochondrial dysfunctions (in particular those triggered by ROS) are typical of neurodegeneration, the role of dietary antioxidants in preventing these multifactorial diseases can be central, and it might pass through the protection of genetic and epigenetic features of the mitochondrial DNA.

Finally, mechanistic aspects of how dietary antioxidants and bioactive molecules interact with the mitochondrial DNA (directly or indirectly either) are outstanding points toward which future research should be directed. Additional data are necessary to put the basis for the identification of specific mitochondrially-targeted DNA that could preserve mtDNA genetics and epigenetics and could be applied in neurodegenerative disorders prevention or treatment (Figure 1).

## 6. Conclusions

Mitochondrial dynamics have been increasingly recognized as a common and early feature likely responsible for neuronal dysfunction in a wide range of neurodegenerative diseases. They strongly depend on ROS production, mtDNA genetics and epigenetics, which act at the interface with environmental exposures. Given the complex interactions between oxidative stress, epigenetics, and mitochondria, it can be postulated that both adequate nutrition [255,256,257] and physical activity [258,259,260] might positively impact the mitochondrial function, and that the effects might be mediated by molecular mechanisms that also include the intervention of epigenetic pathways [169,261]. 

Currently, therapeutic approaches for neurodegenerative diseases are limited and the prevention or alleviation of these multifactorial pathologies is a challenge for contemporary health services. Indeed, early detection and effective protection against neurodegeneration are key factors for a clinical success and healthy aging. Given the relevance of mitochondrial function on neurodegeneration, an epigenetic diet might have both preventive and therapeutic effects, acting at different levels, including the mitochondrial genome. Since the efficiency and number of mitochondria can be manipulated by pharmacological and dietary treatments, it has been suggested that mitochondria may be a potential target for preventive medicine by tailoring personalized dietary approaches and supplementations [174]. Moreover, the emerging usage of mtDNA methylation and copy number as biomarkers [64,65,262,263] emphasizes the relevance of studies on mtDNA genomics and epigenomics, and reinforces the need for further investigations of this aspect, particularly with a focus on neurodegenerative disorders. Further research aimed to test the effects of dietary antioxidants on the mitochondrial genome are warranted.
